# Supplementary material for: Pyroptotic cell-derived factors promote osteoclast differentiation
Source: bioRxiv. 2026 Sep 7:2026.09.03.748049. Preprint. [Version 1] doi: 10.64898/2026.09.03.748049 (PMC13622540; doi:10.64898/2026.09.03.748049)
Supplement: Supplementary file 1 [file media-1.pdf]

Figure S1

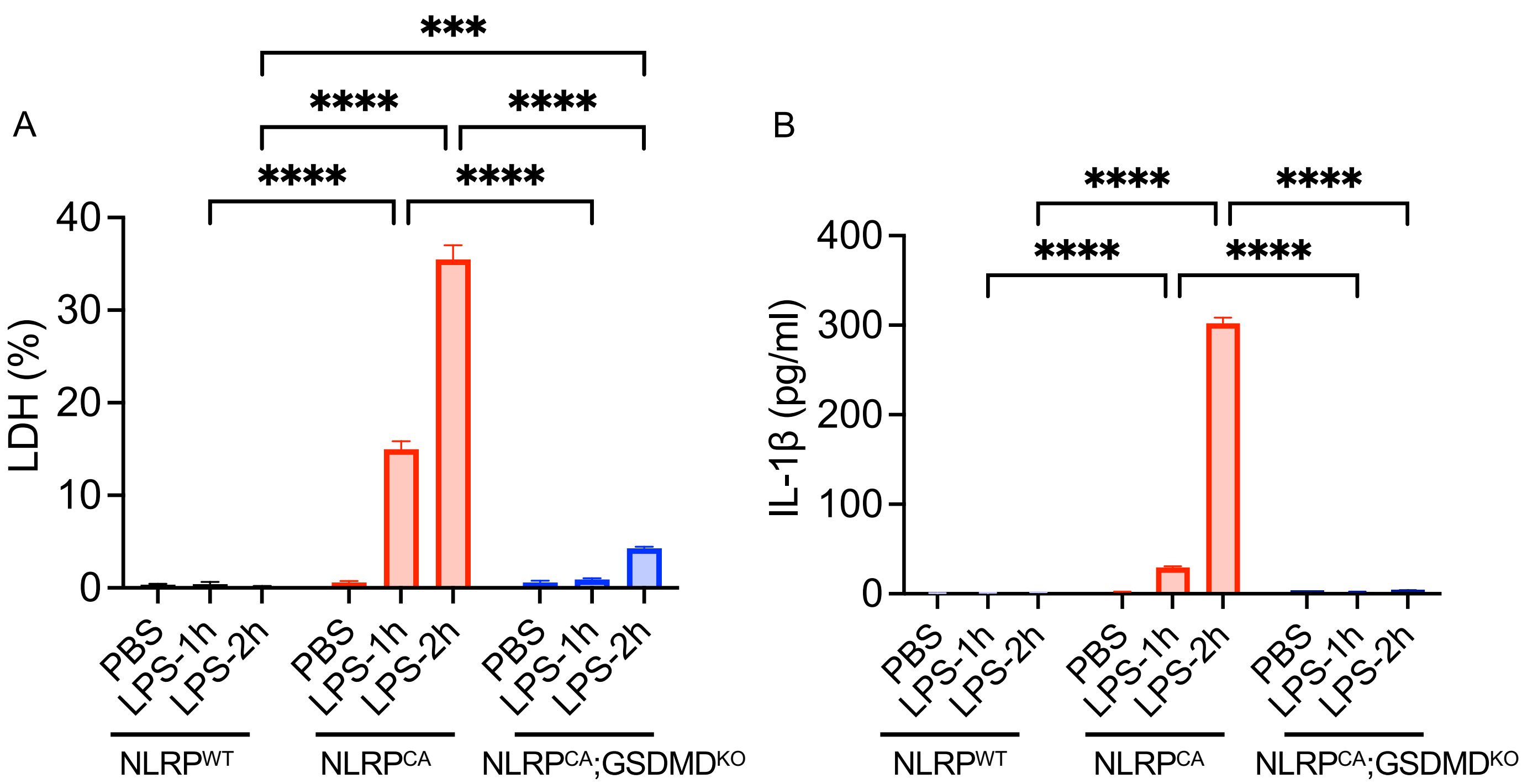

Figure S1. LPS alone causes pyroptosis in NLRP3^{CA}, but not in NLRP3^{WT} or NLRP3^{CA};GSDMD^{KO} BMDMs

BMDMs isolated from NLRP3^{WT}, NLRP3^{CA} and NLRP3^{CA};GSDMD^{KO} mice were treated with PBS or 100 ng/ml LPS for 1 h or 2 h. KO, knockout. Levels of LDH (A) and IL-1β were measured in CM (B). Samples were run in triplicates and data are presented as mean ± SD. ***P<.001, ****P<.0001, two-way ANOVA test.

Figure S2

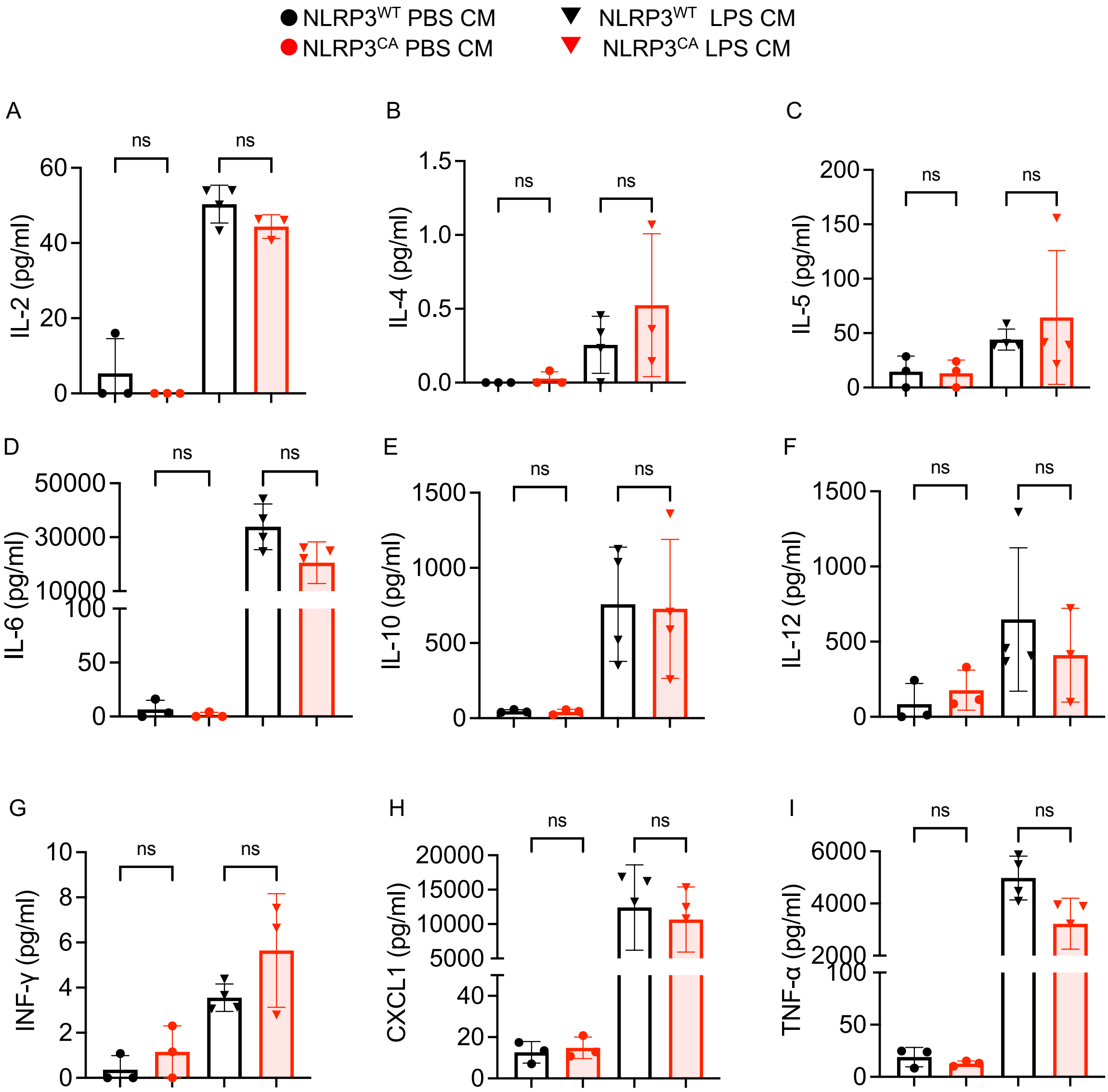

Figure S2. LPS does not induce generalized inflammation through NLRP3^{CA}

The levels of IL-2 (A), IL-4 (B), IL-5 (C), IL-6 (D), IL-10 (E), IL-12 (F), IFN-ɣ (G), CXCR1 (H) and TNF-α (I) were measured in NLRP3^{WT} PBS CM, NLRP3^{CA} PBS CM, NLRP3^{WT} LPS CM. and NLRP3^{CA} LPS CM (n=3~4). Data are presented as mean ± SD. ***P<.001, ****P<.0001; one-way ANOVA test.

Figure S3

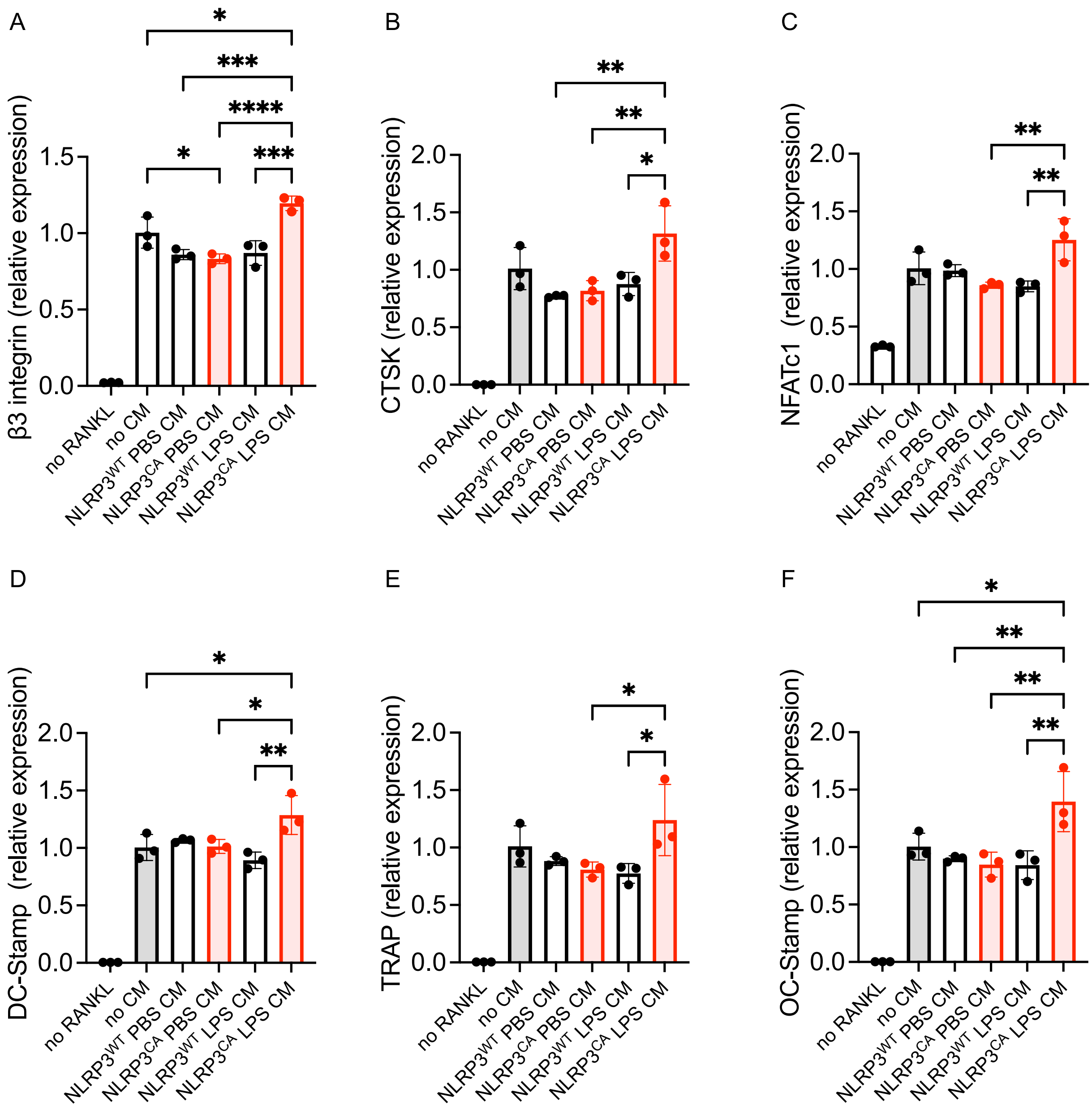

Figure S3. NLRP3^{CA} LPS CM induces stronger expression of OC markers compared to NLRP3^{WT} LPS CM

NLRP3^{WT} PBS CM, NLRP3^{CA} PBS CM, NLRP3^{WT} LPS CM, and NLRP3^{CA} LPS CM were added to WT BMDMs primed with RANKL for 2 days. Cells were cultured for additional 2-3 days in the presence of RANKL and CM refreshed every day. Gene expression was analyzed by qPCR. Samples were run in triplicates and data are presented as mean ± SD. *P<.05, **P<.01, ***P<.001, ****P<.0001; one-way ANOVA test.

Figure S4

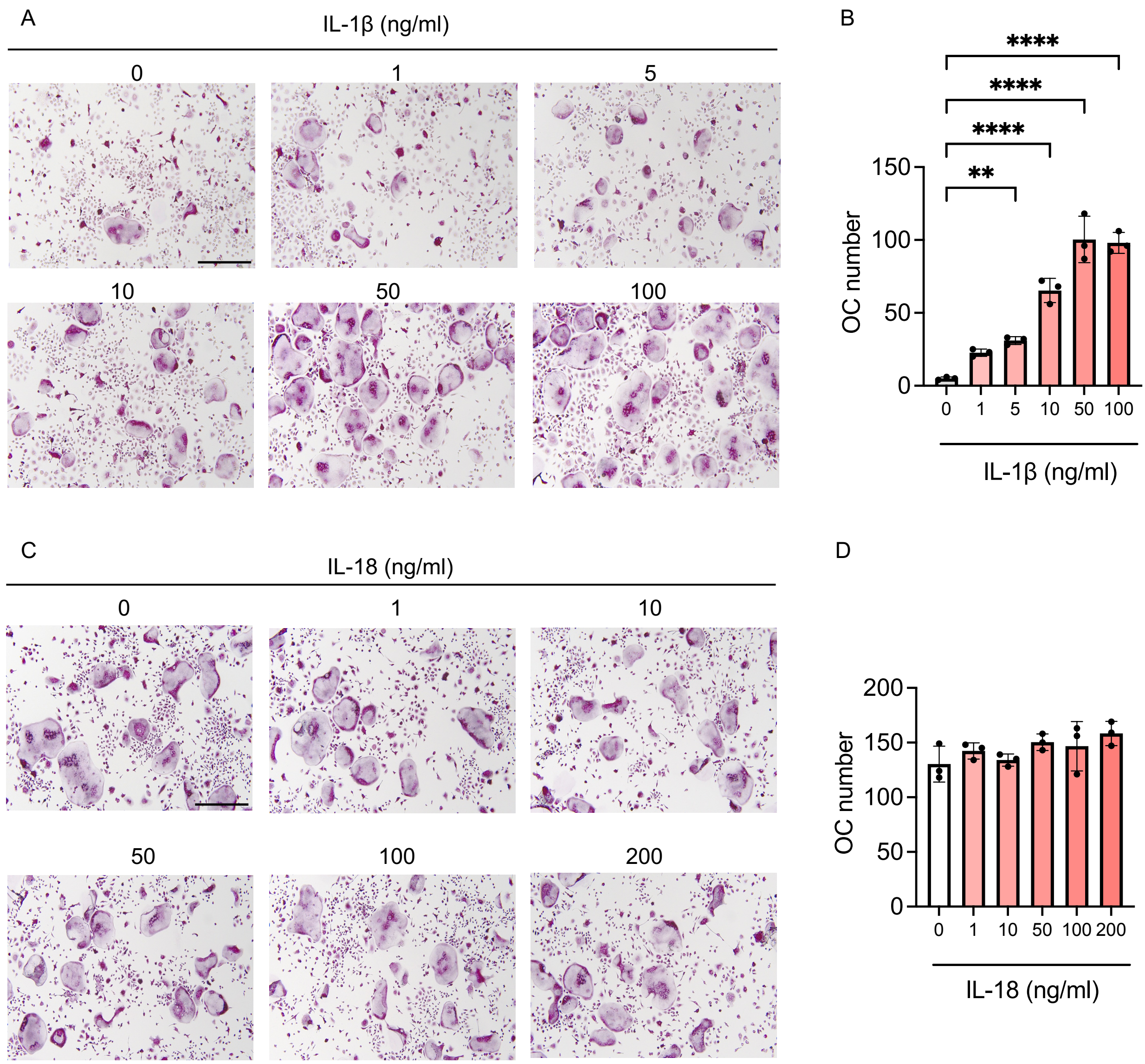

Figure S4. IL-1β but not IL-18 enhances OC differentiation induced by RANKL

IL-1β or IL-18 added to WT BMDMs primed with RANKL for 2 days. Fresh IL-1β or IL-18 was added daily to RANKL-exposed cultures. Cells were stained for TRAP after mature osteoclasts had formed. Representative TRAP-stained images of cells treated with IL-1β (A) or IL-18 (C) in different concentrations, together with quantification of osteoclast numbers (B, D), are shown (n=3). Data are presented as mean ± SD. ***P<.001, ****P<.0001; one-way ANOVA test.

Figure S5

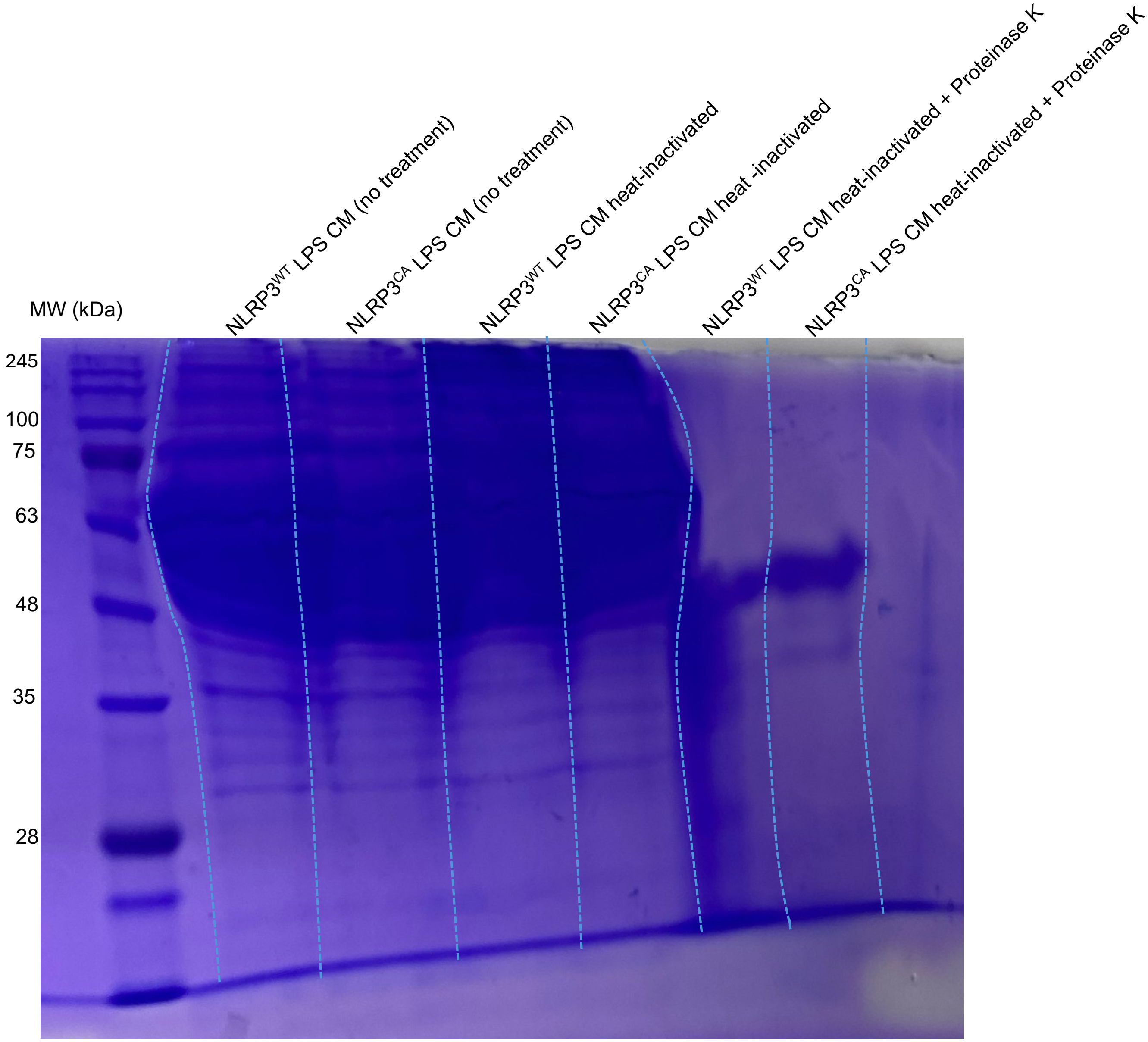

Figure S5. Inactivation of proteins by the heat in the absence or presence of proteinase K

Five µl from untreated NLRP3^{WT} LPS CM, NLRP3^{WT} LPS CM (heat inactivated), NLRP3^{WT} LPS CM (heat inactivated + proteinase K), untreated NLRP3^{CA} LPS CM, NLRP3^{CA} LPS CM (heat inactivated) and NLRP3^{CA} LPS CM (heat inactivated + proteinase K) was subjected to gel electrophoresis, and the gel was stained with Coomassie Brilliant Blue.

Table S1. List of primers for qPCR

Primers	Fw sequence	Rv sequence
Cyclophilin B	AGCATACAGGTCCTGGCATC	TTCACCTTCCCAAAGACCAC
β3 intergrin	TTCGACTACGGCCAGATGATT	GGAGAAAGACAGGTCCATCAAGT
OC stamp	CTGTAACGAACTACTGACCCAGC	CCCAGGCTTAGGAAGACGAAG
DC stamp	ACAAAGCAACAGACTCCCAAAT	GGGGACTTATGTGTTTCCACG
CTSK	AGGCAGCTAAATGCAGAGGGTACA	AGCTTGCATCGATGGACACAGAGA
NFATc1	CCCGTCACATTCTGGTCCAT	CAAGTAACCGTGTAGCTGCACAA
TRAP	CGTCTCTGCACAGATTGCAT	AAGCGCAAACGGTAGTAAGG
